# Regulatory roles of RpoS in the biosynthesis of antibiotics 2,4-diacetyphloroglucinol and pyoluteorin of *Pseudomonas protegens* FD6

**DOI:** 10.3389/fmicb.2022.993732

**Published:** 2022-12-08

**Authors:** Qing Xia Zhang, Zheng Wen Xiong, Shen Yu Li, Yue Yin, Cheng Lin Xing, De Yu Wen, Jian Xu, Qin Liu

**Affiliations:** ^1^College of Plant Protection, Yangzhou University, Yangzhou, China; ^2^College of Resources and Environmental Sciences, Nanjing Agricultural University, Nanjing, China; ^3^Jiangsu Lixiahe District Institute of Agricultural Sciences, Yangzhou, China

**Keywords:** *Pseudomonas*, biocontrol agent, RpoS, antibiotic biosynthesis, stress resistance

## Abstract

The rhizosphere microbe *Pseudomonas protegens* FD6 possesses beneficial traits such as the production of antibiotics like pyoluteorin (Plt) and 2,4-diacetylphloroglucinol (2,4-DAPG). The alternative RpoS (σ^38^ factor), as a master regulator, activates or inhibits the transcription of stationary phase genes in several biocontrol organisms. Here, we investigated the complicated function and regulatory mechanism of RpoS in the biosynthesis of 2,4-DAPG and Plt in strain FD6. Phenotypic assays suggested that ΔrpoS was impaired in biofilm formation, swimming motility, swarming motility, and resistance to stress, such as heat, H_2_O_2_ and 12% ethanol. The RpoS mutation significantly increased both 2,4-DAPG and Plt production and altered the transcription and translation of the biosynthetic genes *phlA* and *pltL*, indicating that RpoS inhibited antibiotic production by FD6 at both the transcriptional and translational levels. RpoS negatively controlled 2,4-DAPG biosynthesis and transcription of the 2,4-DAPG operon *phlACBD* by directly interacting with the promoter sequences of *phlG* and *phlA.* In addition, RpoS significantly inhibited Plt production and the expression of its operon *pltLABCDEFG* by directly binding to the promoter regions of *pltR, pltL and pltF.* Further analyzes demonstrated that a putative R147 mutation in the RpoS binding domain abolished its inhibitory activity on the expression of *pltL* and *phlA*. Overall, our results reveal the pleiotropic regulatory function of RpoS in *P. protegens* FD6 and provide the basis for improving antibiotic biosynthesis by genetic engineering in biocontrol organisms.

## Introduction

*Pseudomonas* spp., a group of more than 100 species, are widely abundant in soil and play an important role in the rhizosphere (Mulet et al., 2010). This group is well known as an effective biological control bacterium in various hosts, reducing disease incidence incited by air-borne plant pathogens and soil-borne phytopathogenic fungi (Ligon et al., 2000). The most common mechanisms of biological control by pseudomonades include the production of lytic exoenzymes, hydrogen cyanide (HCN), siderophores, cyclic lipopeptides and antibiotics. The antibiotics pyrrolnitrin (Prn), pyoluteorin (Plt), phenazine (Phz) and 2,4-diacetylphloroglucinol (2,4-DAPG) have been revealed as the main antifungal biocontrol substances produced by *Pseudomonas* spp. (Mishra and Arora, 2018; Keswani et al., 2020).

Some *Pseudomonas* spp., including *P. protegens* H78, Pf-5, FD6 and CHA0, produce both 2,4-DAPG and Plt, which is possibly involved in biological control (Bottiglieri and Keel, 2006; Yan et al., 2017a; Liu et al., 2018b; Zhang et al., 2020). The biosynthetic locus of 2,4-DAPG consists of four structural genes, *phlACBD*, and several modifying genes (such as *phlF* and *phlH*) that are involved in the synthesis of 2,4-DAPG and its precursors. These four genes *phlACBD* transcribed together and constitute an operon (Bangera and Thomashow, 1999). PhlG is specifically involved in the degradation of 2,4-DAPG to monoacetylphloroglucinol, which displays less toxicity against phytopathogens (Bottiglieri and Keel, 2006). The 2,4-diacetylphloroglucinol functioning as an autoinducer stimulates the transcription of the 2,4-DAPG locus (Schnider-Keel et al., 2000). The synthesis of 2,4-DAPG initiates with the biosynthesis of phloroglucinol (PG) from three molecules of malonyl-coenzyme A by PhlD (Achkar et al., 2005). PhlABC acetylates PG to form 2,4-DAPG (Bangera and Thomashow, 1999). The Plt biosynthesis gene cluster consists of the structural gene *pltLABCDEFG*, transcriptional regulators *pltR* and *pltZ*, and transporter operon *pltIJKNOP*. PltR is a transcriptional activator and transcribes in a direction opposite to the *pltLABCDEFG* operon (Nowak-Thompson et al., 1999). Recently, it has been reported that chlorinated phloroglucinols, intermediates in 2,4-DAPG biosynthesis, function as cellular signals to induce pyoluteorin production (Yan et al., 2017a). In addition, the synthesis of both 2,4-DAPG and Plt is typically controlled by a variety of complex regulatory systems, such as the GacA/GacS two–component system, quorum sensing and sigma factors (Dwivedi and Johri, 2003).

In bacteria, σ-factor is necessary for the RNA polymerase core enzyme to recognize gene promoters for transcription initiation. The stationary phase sigma σ^38^ (RpoS) belongs to the sigma 70 family and was originally defined in *Escherichia coli* as a global regulator that modulates approximately 23% genes from the *E. coli* genome (Hengge-Aronis, 2000; Wong et al., 2017). Regarded as a master regulator, σ^38^ is involved in stress resistance, virulence and the production of antifungal metabolites in many gram-negative bacteria (Liu et al., 2019; Qin et al., 2020). In *Pseudomonas* species, RpoS was found to have an effect on secondary metabolism, particularly on antibiotic production (Suh et al., 1999; Yan et al., 2009). However, the results vary according to the species and the antifungal metabolites in question. For example, a defect in *rpoS* not only decreased pyrrolnitrin production by *P. fluorescens* but also resulted in excess synthesis of pyoluteorin and 2,4-diacetylphloroglucinol (Sarniguet et al., 1995). However, a mutation in *rpoS* leads to a decrease in phenazine-1-carboxamide production in *P. chlororaphis* PCL1391 (Girard et al., 2006). Therefore, the different regulatory mechanism of RpoS in antibiotic biosynthesis is still unknown. RpoS consists of four functional domains, and the most highly conserved 2 domain is crucial for recognition of the-10-promoter region (Lévi-Meyrueis et al., 2015). RpoS recognizes a consensus-10 box sequence (CTATACT) in *P. aeruginosa;* however, no distinctive-35 box sequence motif has been described for *Pseudomonas* spp. (Schuster et al., 2004; Hall et al., 2018).

*P. protegens* FD6 (previously named *P. fluorescens*) has been reported to simultaneously produce Plt, 2,4-DAPG and Prn, which efficiently inhibit airborne phytopathogenic fungi and soil-borne plant pathogens (Chang et al., 2011; Zhang et al., 2021a). Our previous studies showed that these three antibiotics were negatively controlled by the hybrid sensor kinase RetS and the virulence factor regulator Vfr (Zhang et al., 2015, 2016). Furthermore, Vfr may directly bind to promoter regions of the *phlG* and *phlF* (Zhang et al., 2021b). *P. protegens* FD6 shows strong antifungal activities, mainly due to the biosynthesis of antibiotics such as 2,4-diacetylphloroglucinol and pyoluteorin (Zhang et al., 2020). The regulatory function of RpoS has been reported in many bacteria, the repression mechanism by which RpoS controls the biosynthesis of Plt and 2,4-DAPG in pseudomonades remains unclear. The objects of this paper were to reveal the regulatory role of RpoS in the strain FD6 and to perform a bioinformatic search to locate its possible target genes that could clarify the regulatory function of the RpoS protein. In addition, our point mutation experiments demonstrated that DNA binding to RpoS is necessary to repress the expression of *phlA* and *pltL*. These results suggest that RpoS plays a negative role in the production of 2,4-diacetylphloroglucinol and Plt by binding to the upstream sequences of the *phlA*, *phlG, pltR*, *pltL* and *pltF* genes and positively regulating motility, stress resistance and biofilm formation.

## Materials and methods

### Strains, plasmids and growth conditions

The plasmids and bacterial strains used in this study are described in Supplementary Table S1. *P. protegens* FD6 was originally isolated from the canola rhizosphere (Chang et al., 2011). The complete genomic DNA of FD6 is available in the NCBI GenBank (accession no. CP031396; Zhang et al., 2020). All strains were routinely cultivated on Luria-Bertani (LB) medium at 28°C. *Escherichia coli* DH5α was grown on LB at 37°C. Antibiotics were added as follows: ampicillin 50 μg/ml, kanamycin 50 μg/ml, and tetracycline 20 μg/ml for *E. coli* or 30 μg/ml for *P. protegens* strains, gentamycin 10 μg/ml for *E. coli* or 30 μg/ml for *P. protegens* strains.

### DNA manipulation and sequence analysis

The chromosomal DNA of *P. protegens* FD6 was isolated using the CTAB method as described previously (Sal et al., 1988). Small-scale plasmid preparations were performed using a kit (Axygen, Corning Life Sciences China). Standard techniques for restriction, PCR amplification, gel extraction and transformation were performed following standard protocols (Sambrook and Russell, 2001). Nucleotide sequencing was carried out by Nanjing Qing Ke Biological Technology Co., Ltd. (Nanjing, China).

### Mutant construction

An *rpoS* in-frame deletion mutant was constructed by homologous recombinant. All PCRs were performed using PrimeSTAR Max DNA polymerase (TaKaRa, Japan). To generate the *rpoS* mutant, a 762-bp fragment was deleted in frame from the *rpoS*. The 1,209 bp and 674 bp fragments were amplified from chromosomal DNA of the wild-type (WT) strain of *P. protegens* FD6 by PCR using primers ropS422-F1/ropS1695-R1 and ropS2482-F2/ropS3138-R2. In the second round of PCR, 20 ng of the above PCR products was used as a template for the third round of amplification using primers ropS422-F1 and ropS3138-R2.

The fusing fragment was digested with *Xba*I and *Sac*I and cloned into the suicide vector p2P24 (Yan et al., 2017b). All constructs were transformed into *E. coli* DH5α and *E. coli* S17-1 (Simon et al., 1983). Biparental matings were performed with the WT strain and transconjugants selected on LB with ampicillin and kanamycin. The ΔrpoS mutation was verified by PCR and sequencing. The *ropS* gene with its intact promoter was amplified with the primers ropS1239-F and ropS2650-R (Supplementary Table S2) and inserted into the wide-host vector pBBR1MCS-2 to generate pBBR-ropS for the complementation of the ΔropS mutant.

The *rpoS* allele in *P. protegens* FD6 was replaced by rpoSR147S *via* PCR site-directed mutagenesis. Briefly, the 1,008 bp *rpoS* PCR product was cloned into the pMD19-T vector to generate pMD-rpoS using the primers RpoS M-F and RpoS M-R. The R147S mutation was obtained from pMD-rpoS by PCR amplification with the primers RpoS-mut-F and RpoS-mut-R. After 35 cycles, the PCR products were digested with *Dpn*I (Takara, Japan) to remove the parental plasmid and then purified with the Biospin PCR Purification Kit (Bioer Technology, China). The purified plasmids were transformed into *E. coli* DH5α to generate pMD-rpoS_R147S_, which was verified by DNA sequencing. The *rpoS* point mutation was amplified from pMD-rpoS_R147S_ plasmid DNA by PCR using primers RpoS-mut-pBBR-F and RpoS-mut-pBBR-R and cloned into the *Sal*I and *Xba*I sites of the pBBR1MCS-2 to generate pBBR-rpoS_R147S_. This *rpoS*R147S mutation was introduced in ΔrpoS to generate the rpoS_R147S_ mutant using electroporation for subsequent study.

### Determination of the growth curve

Overnight bacterial suspensions were adjusted to an OD_600_ of 0.5, and shaken at an agitation speed of 180 rpm under. 28°C with 180 rpm spin speed. The OD_600_ was measured every 3 h throughout the growth period. Each strain was tested with three replicates.

### Construction of *lacZ* transcriptional fusions and β-galactosidase activity assays

To assay the regulatory functions of RpoS on 2,4-DAPG and Plt, the promoter regions of *phlA* and *pltL* were amplified with primers phlA6013-F-440/phlA6013-R27 and pltL6013-57F/ pltL6013-616R (Supplementary Table S2) and cloned into the pMD19-T vector. The recombinant plasmids were digested with *Pst*I/*Eco*RI and cloned separately into the pME6522 plasmid ahead of a promoterless *lacZ* gene. The plasmid pME6013 with a tetracycline resistance marker was used to construct the *lacZ* translational fusions.

β-Galactosidase activities in *P. protegens* harboring the *lacZ* reporter gene were assayed by the Miller method (Miller, 1972). β-Galactosidase activities in *P. protegens* derivatives harboring the pME6522 empty vector were subtracted. All measurements were performed after inoculation of 20 ml LB with 20 μl overnight cultures and incubation at 28°C with constant shaking.

### Phenotypic analysis of the RpoS mutant

Extracellular protease and biofilm formation were tested as described previously (Reimmann et al., 1997; O'Toole et al., 1999). To test swimming and swarming abilities, MMMF and SWM agar plates were used for the swimming and swarming tests, respectively (Taguchi and Ichinose, 2013).

### Stress response assays

Stress response was assessed as previously described (Sarniguet et al., 1995). Overnight cultures in LB broth were centrifuged, suspended in M9 liquid medium, and diluted to OD_600_ = 0.5. The suspensions supplemented with 12% ethanol and 5 M H_2_O_2_ were cultured at 28°C for 15 min for ethanol and oxidative challenges. The suspensions supplemented with 2.4 M NaCl at 28°C for 45 min for the osmotic challenge, and cultured at 45°C for 15 min for the high-temperature challenge.

### Detection of antimicrobial activity

Radial diffusion assays were carried out to detect antifungal activity *in vitro* as described previously (Zhang et al., 2020). A 5 mm plug of *Monilinia fructicola*, *Phytophthora capsici* or *Botrytis cinerea* was transferred to the center of a potato sucrose agar (PSA) plate, and 5 μl of overnight cultures was dropped onto the edge of the plate. The plates were placed at 25°C, and antimicrobial activity was determined after 5–10 d by gauging the width of the inhibitory zone. Similarly, to detect antagonistic activity against *Ralstonia solanacearum*, 3 μl overnight cultures of FD6 and its derivatives were spotted onto a TM (0.5 g/l beef extract, 10 g/l glucose, 5 g/l tryptone and 3 g/l yeast extract) plate that was already mixed with *R. solanacearum*. The plate was placed at 28°C, and inhibitory activity was detected 2 days later.

### Quantification of Plt and 2,4-DAPG production

The quantification of 2,4-DAPG and Plt were performed *via* HITACHI L-2000 HPLC using a C18 reverse-phase column as previously described (Zhang et al., 2015). Three replicates were used for each treatment. The purified products Plt and 2,4-DAPG were selected as controls.

### qRT–PCR analysis

qRT–PCR procedures were performed as described previously, and the primer sequences are listed in Supplementary Table S2 (Zhang et al., 2015, 2020). The *rrsB* transcript used as an internal control for real-time PCR (Zhang et al., 2015). Experiments were carried out in three independent biological replicates. Relative fold change of each genes was determined according to the 2^-ΔΔCt^ method (Livak and Schmittgen, 2001).

### Bacterial one-hybrid assays

A bacterial one-hybrid system was reported in our recent report and used to determine potential interactions between RpoS protein and its target DNA (Zhang et al., 2021b). The possible RpoS binding sequences were cloned into pBXcmT. The RpoS open reading frame was cloned into the plasmid pTRG, resulting in the recombinant construct pTRG-rpoS (Supplementary Table S1). The positive cotransformant pTvfr-pBvfr was selected as a positive control as described recently (Zhang et al., 2021b). Transformant carrying empty pBXcmT and pTRG vectors was utilized as a negative control. All cotransformants were dropped onto selective media and cultured at 30°C for 4–5 days. The possible interactions between RpoS and its target gene promoters were identified on the selective medium described in a previous report (Xu et al., 2016). This nonselective medium was used to confirm that both vectors were successfully introduced into the target *E. coli* XL1-Blue MRF9 Kan strains.

### RpoS purification

For the overexpression of RpoS with a C-terminal fused His-tag, the oligonucleotides RpoS-NdeI-F and RpoS-XhoI-R were used to amplify the *rpoS* coding sequence. The PCR product was digested with *Xho*I and *Nde*I and cloned into the pET22b (+) to give pET22b-rpoS. The recombinant RpoS protein was expressed from pET22b-rpoS in *E. coli* BL21 (DE3). After induction with 1 mM IPTG at 16°C for 6 h, cells were collected and resuspended in suspension buffer (500 mM NaCl, 10% glycerol, 15 mM Tris–HCl, pH 6.5). After cells were sonicated with a JY92-IIDN Cell Disrupter, the soluble expressed protein was loaded onto High Affinity Ni-NTA Resin (GenScript), and purified following the manufacturer’s protocol. The recombinant His-RpoS protein was eluted by 200 mM imidazole, and verified by SDS-PAGE. The concentration of the purified RpoS protein was assayed using the BCA Protein Assay Kit.

### Electrophoretic mobility shift assays (EMSAs)

An EMSA was performed *via* the Beyotime EMSA/Gel-Shift kit (No. GS002) and the Chemiluminescent EMSA Kit (No. GS009) as recently described, with minor modifications (Zhang et al., 2021b). Binding assays were carried out by incubating 1 μmol chemiluminescent-labeled probes (Supplemental Table S2) with increasing amounts of purified recombinant RpoS. Competition experiments were performed with 100-fold cold probes (not biotin labeled). Free DNA and protein-bound DNA were applied to a 4% nondenaturing polyacrylamide gel in 0.5 × Tris-borate-EDTA buffer, pH 8.0. After electrophoresis, gels were blotted onto nylon membranes and crosslinked as described in the manufacturer’s instructions. A chemiluminescence detection method was utilized to determine the biotinylated DNA fragments and analyzed by Image Lab™ (BIO-RAD, United States).

### Statistical analysis

The statistical analysis was performed using Student’s *t* test implemented in the GraphPad Prism software. In the present study, all experiments were carried out twice independently. Data are expressed as the average ± SD (standard deviation) of three replications.

## Results

### Analysis of phenotypic characterization

To assay whether the growth of ΔrpoS was defective, the growth proficiency of ΔrpoS was compared with that of *P. protegens* FD6 using LB medium. The growth curves of these three strains suggested that the ΔrpoS was less than that of WT FD6 from 6 to 48 h, while the complementary strain ΔrpoSC could restore the growth rate to near wild-type levels (Supplementary Figure S1). The biofilm assay showed that the *rpoS* mutant had a hypobiofilm comparable to strain FD6 and its complementary strain ΔrpoSC, suggesting that RpoS positively regulates biofilm formation in *P. protegens* FD6 (Supplementary Figure S2A). The phenotypic characterization showed that both the swimming and swarming motilities of ΔrpoS decreased significantly, suggesting that the motility of ΔrpoS is impaired (Supplementary Figure S2B).

### RpoS is essential for stress resistance

**Figure 1:**
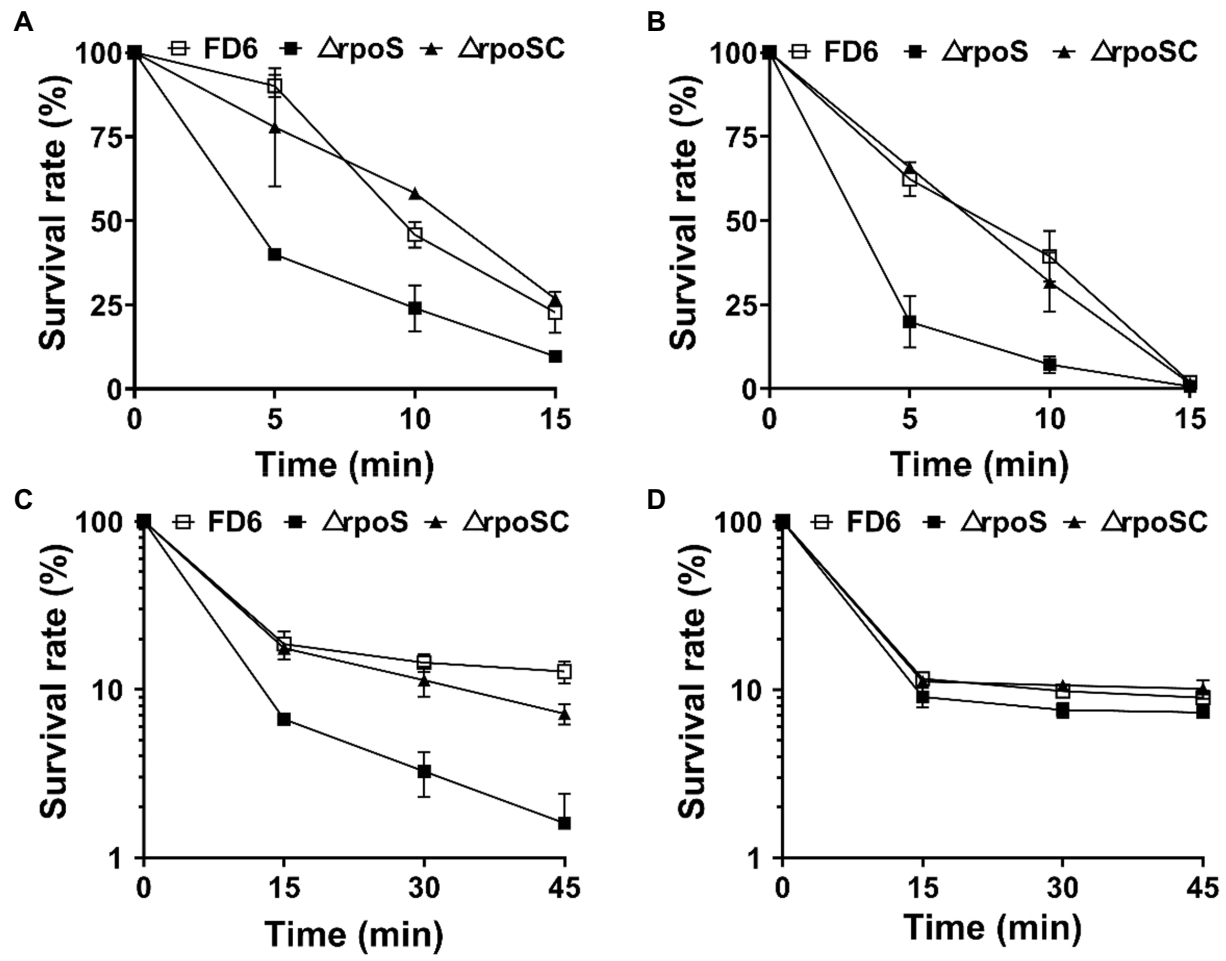
Roles of *rpoS* in resistance to **(A)** heat, **(B)** 12% ethanol, **(C)** 5 M H_2_O_2_ or **(D)** 2.4 M NaCl. Cultures of *Pseudomonas protegens* FD6 strains were exposed to different stress conditions. The bacterial cells were resuspended in sterilized M9 liquid medium to test cell viability. The experiment was carried out in three biological replicates. Error bars represent ±standard deviation.

The survival rates of wild-type FD6, ΔrpoS and the complementary strain ΔrpoSC in the stationary phase were assayed after exposure to diverse stress conditions, including 45°C, 12% ethanol, 5 M H_2_O_2_ and 2.4 M NaCl (Figure 1). The survival rate of ΔrpoS cells exposed to 45°C or 12% ethanol was significantly lower than that of wild-type cells (Figures 1A,B). After exposure for 10 min, the survival rates of FD6 were 1.9 and 5 times that of ΔrpoS, respectively. After treatment with 5 M H_2_O_2_ for 45 min, the survival rate of FD6 was 7.5 times that of ΔrpoS mutant (Figure 1C). For the osmotic stress (2.4 M NaCl), the survival rates of ΔrpoS is similar to that of the wild type (Figure 1D).

### Effect of RpoS on antimicrobial activities in *Pseudomonas protegens* FD6

**Figure 2:**
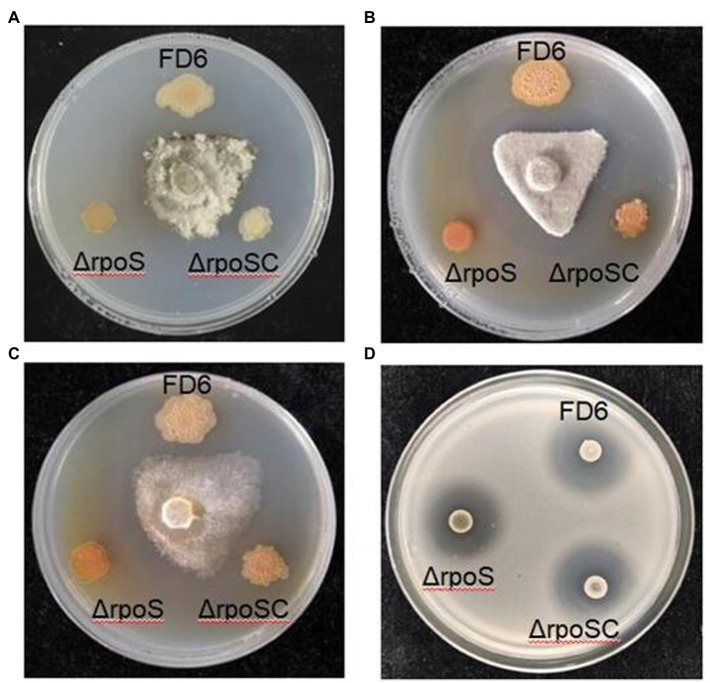
Effect of RpoS on the antagonistic ability of *P. protegens* FD6 against **(A)** *Botrytis cinerea*, **(B)** *Monilinia fructicola*, **(C)** *Phytophthora capsici* and **(D)** *Ralstonia solanacearum*. The antifungal abilities of FD6 and its derivatives were determined by measuring the inhibition zone of these phytopathogenic fungi on PSA plates. The antibacterial activities of FD6 and its derivatives against *R. solanacearum* were assessed on TM plates. The experiment was performed in three biological replicates.

Four phytopathogens, *B. cinerea*, *M. fructicola*, *P. capsici* and *R. solanacearum,* were chosen to determine the effect of RpoS on the antimicrobial activity of *P. protegens* FD6. Mutation of the *rpoS* gene (ΔrpoS) resulted in increased inhibitory ability (1.78-, 1.42-and 2.53-fold in *B. cinerea*, *M. fructicola* and *P. capsici*) compared with wild-type FD6. Furthermore, the color of the colonies darkened, which suggests that more specific metabolite production was associated with stronger antifungal activity (Figures 2A–C). The RpoS mutation also caused stronger antimicrobial activity of FD6 against *R. solanacearum* and produced a clear inhibition zone (Figure 2D).

### Negative control of *phlACBD* operon expression and 2,4-DAPG biosynthesis by RpoS

**Figure 3:**
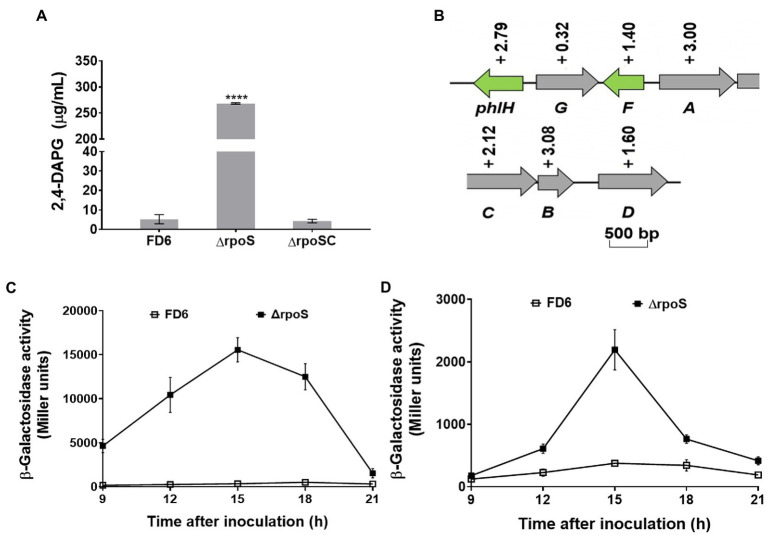
Negative modulation of 2,4-DAPG synthesis and operon expression by RpoS. **(A)** 2,4-DAPG biosynthesis was quantified using HPLC. **(B)** Effect of RpoS on the transcript level of the 2,4-DAPG synthesis genes in *Pseudomonas protegens* FD6. Gray and green indicate that these genes are involved in biosynthesis and regulation, respectively. **(C,D)** Deletion of *rpoS* significantly increased the expression of the *phlA-lacZ* and *phlA′-′lacZ* fusions. Error bars show mean ± standard deviation. ****p < 0.0001.

The synthesis of 2,4-diacetylphloroglucinol was significantly increased in the rpoS-deleted strain. 2,4-DAPG production was elevated over 50 times in ΔrpoS (Figure 3A). The *rpoS* deletion mutant created a 1.40-to 3.08-fold increase in the transcriptional levels of most genes in the 2,4-DAPG biosynthetic gene cluster *phlHGFACBD*, as determined by qRT–PCR (Figure 3B). The β-galactosidase assays of the *phlA-lacZ* and *phlA′-′lacZ* fusions further confirmed that the expression of the *phlACBD* biosynthesis gene clusters was negatively regulated by RpoS at both the transcriptional and translational levels (Figures 3C,D).

### Negative control of pyoluteorin gene cluster expression and Plt production by RpoS

**Figure 4:**
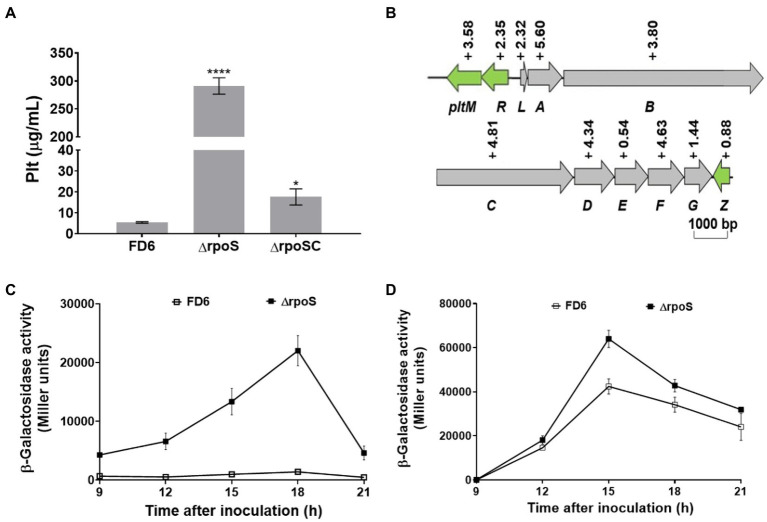
Negative regulation of Plt production and the expression of gene cluster by RpoS. **(A)** HPLC analysis of Plt biosynthesis by FD6 and its derivatives. **(B)** Influence of RpoS on the transcript level of the Plt biosynthesis gene cluster in *Pseudomonas protegens* FD6. Gray and green indicate the same meanings. **(C,D)** Deletion of *rpoS* markedly increased the expression of the *pltL-lacZ* and *pltL′-′lacZ* fusions. Error bars represent the standard deviation of the mean. *p < 0.05, and ****p < 0.0001.

The RpoS deletion mutation also led to the production of pyoluteorin being markedly enhanced by over 50-fold compared with the WT, as determined by HPLC (*p* < 0.01; Figure 4A). The complementary strain ΔrpoSC diminished significantly the level of Plt, whereas there is significant difference between ΔrpoSC and FD6. This showed that RpoS has a negative effect on the Plt production. The expression of the Plt gene cluster is negatively controlled by RpoS in the *rpoS* deletion mutant compared to *P. protegens* FD6. The *rpoS* deletion induced a 0.54- to 5.60-fold higher expression in the *pltMRLABCDEFGZ* operon (Figure 4B). Furthermore, the β–galactosidase data indicated that the *rpoS* mutation had a negative influence on *pltL* expression at both the transcript and translation levels (Figures 4C,D).

### Confirmation of RpoS-DNA binding and the target sequence

**Figure 5:**
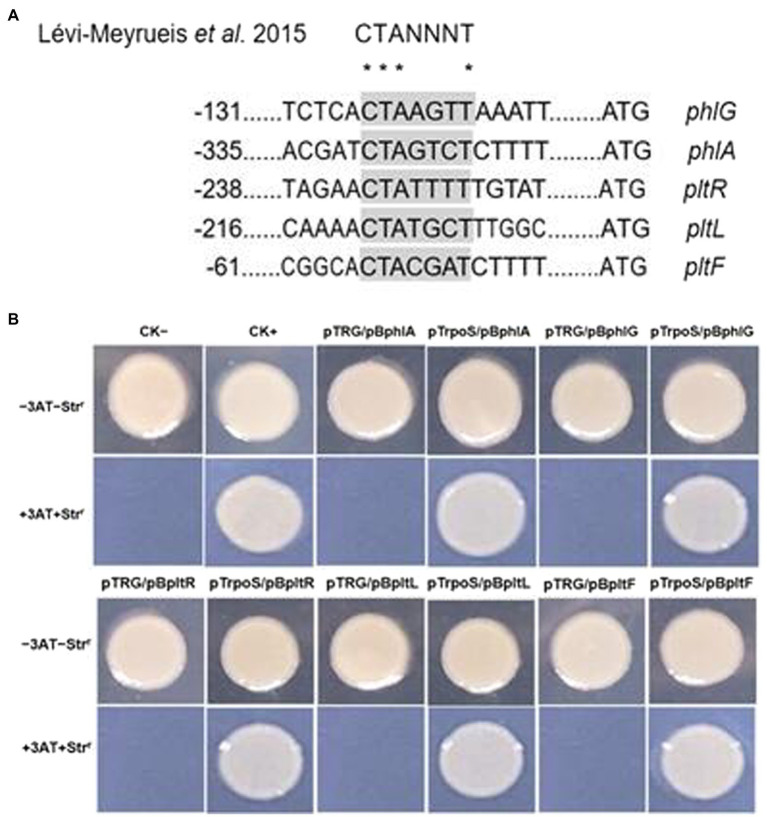
RpoS and its target DNA-binding specificity assay. **(A)** Identification of the RpoS-10 consensus motif in the promoter regions of antibiotic biosynthetic genes in *P. protegens* FD6. **(B)** Direct interactions between RpoS with the promoter regions of its targets in *P. protegens* FD6. The *E. coli* XL1-Blue MRF’ Kan carrying pTvfr and pBvfr plasmids were used as a positive control on a selective screening medium plate.

RpoS usually binds to the promoter sequence of its target genes. Lévi-Meyrueis et al. (2015) described the-10 RpoS binding motif as CTANNNT. To clarify the function of RpoS in antibiotic synthesis, we used the possible consensus sequences recognized by RpoS to search for putative target genes and identified several genes encoding 2,4-diacetylphloroglucinol and pyoluteorin biosynthetic gene clusters in the *P. protegens* FD6 genome (NZ_CP031396.1). Interestingly, we found that 2 promoter regions of the 2,4-DAPG operon and 3 promoter regions of the Plt operon contain the sequence CTANNNT, which closely coincides with the-10 element for RpoS binding (Figure 5A). To detect the interactions between protein-DNA, we first performed bacterial one-hybrid experiments. Our previous study demonstrated that Vfr could bind to its own promoter. Therefore, we used the cotransformant with pTvfr/pBvfr as a positive control to test the above RpoS possible target genes *via* bacterial one-hybrid system assays. As displayed in Figure 5B, the strains with pTrpoS/pBphlA, pTrpoS/ pBphlG, pTrpoS/pBpltR, pTrpoS/pBpltL or pTrpoS/pBpltF grew well which is similar to the pTvfr/pBvfr. Furthermore, those strains with pTRG/pBphlA, pTRG/pBphlG, pTRG/pBpltR, pTRG/pBpltL or pTRG/pBpltF failed to grow. We conclude that RpoS specifically binds with the promoter regions of *phlA*, *phlG*, *pltR*, *pltL* and *pltF*.

### RpoS-DNA interactions determined with the EMSA

**Figure 6:**
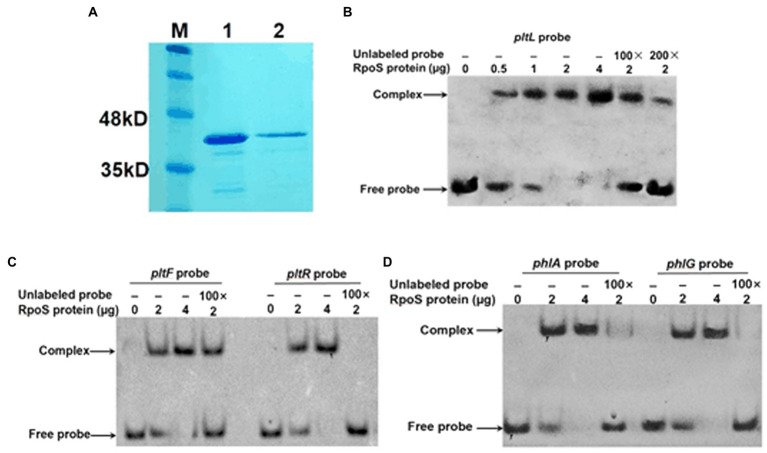
Direct interaction of RpoS with the promoter regions of antibiotic biosynthesis clusters. **(A)** Purification of the RpoS protein. M: Protein standard marker; 1–2 the pure RpoS (protein eluted with 200 mM and 250 mM imidazole). **(B–D)** An electrophoresis mobility shift assay was used to evaluate the interaction between RpoS and labeled DNA probes. The amount of RpoS used in this experiment is shown above each gel. The concentration of labeled probe was 1 μM. An excess of unlabeled probe was used in the competitive experiment.

To confirm whether RpoS repressed the above five genes through direct binding, we expressed and purified the RpoS protein fused with a C-terminal His-tag and then purified it with Ni-NTA resin (Figure 6A). The concentration of RpoS was 846 μg/ml according to the BCA Protein Assay Kit. We performed an EMSA with purified RpoS and synthesized 25-bp DNA probes labeled with biotin. The EMSA revealed that RpoS bound to all five DNA probes to form protein–DNA complexes (Figures 6B–D), which is also in agreement with the bacterial one-hybrid data. Moreover, the RpoS–DNA complexes revealed stronger retarded bands with increasing amounts of the RpoS protein. The RpoS–DNA complexes disappeared or weakened, when unlabeled competitors were present (Figures 6B–D). These results suggest that these affinities of RpoS to DNA are highly specific.

### DNA binding is necessary for the RpoS-mediated inhibition of *phlA* and *pltL* expression

**Figure 7:**
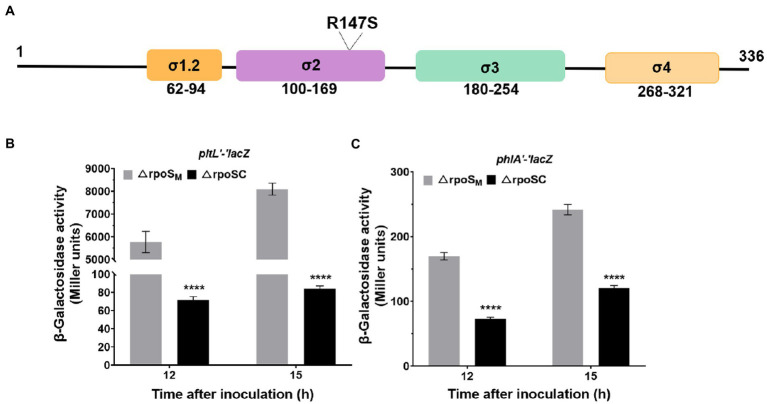
Effect of ΔrpoS on the expression of *phlA′-′lacZ* and *pltL′-′lacZ*. **(A)** Schematic representation of the four regions of the RpoS protein and location of the R147 mutation in region 2, which binds to the-10 promoter of a target gene. **(B,C)** Differences in β-galactosidase expression in two reporter vectors, 6,013-pltL and 6,013-phlA, were determined between *P. protegens* FD6 derivatives grown for 12 h and 15 h in LB at 28°C. The plasmid pBBR1MCS-2, carrying the *rpoS* or *rpoS*_R147S_ genes, was used in complementation experiments. The experiment was performed in three biological replicates. Error bars indicate ±standard deviation. ****p < 0.0001.

The RpoS protein is composed of four functional regions: σ1.2, σ2, σ3 and σ4 (Figure 7A). The σ2 region is crucial for recognition of the-10 promoter element, which is the essential and most highly conserved promoter motif (Lévi-Meyrueis et al., 2015). Amino acid R147 was substituted for S147 in the σ2 region, which binds to the-10 promoter element of target genes (Figure 7A). To further explore the molecular mechanism by which RpoS affects 2,4-DAPG and Plt biosynthesis, RpoS-inhibited *lacZ* fusions relating to the 2,4-DAPG and Plt operons were examined. Expression of the *phlA′-′lacZ* and *pltL′-′lacZ* translational fusions was also upregulated in the ΔrpoS_M_ mutant compared to the complementary strain ΔrpoSC at 12 and 15 h. In particular, the ΔrpoS_M_ mutant induced a significant increase in the translational levels of *pltL* genes compared to strain ΔrpoSC (Figures 7B,C). Genetic analysis suggested that DNA binding of RpoS is required for suppressing 2,4-DAPG and Plt biosynthesis.

## Discussion

**Figure 8:**
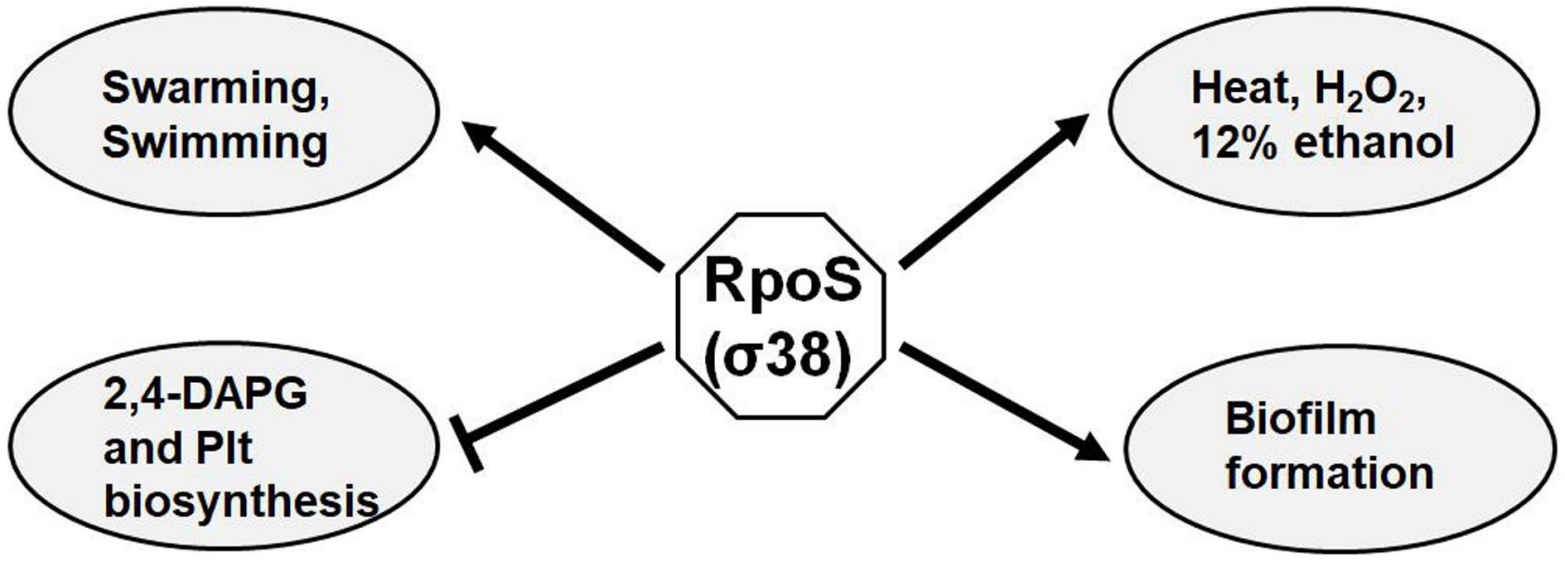
Proposed regulatory model of RpoS on biocontrol factors in *P. protegens* FD6. Arrows, positive regulation; lines with a flattened end, negative regulation.

Sigma factors are ubiquitous in bacterial genomes. The *P. fluorescens* Pf-5 genome, which shares 98.7% similarity of nucleotide sequences with that of *P. protegens* FD6, has a total of 33 sigma factors, including one σ^54^ and four σ^70^ (Kill et al., 2005; Zhang et al., 2020). The sigma factor RpoS has been reported as a global regulator of the cell functions in many gram-negative bacteria, including stress resistance, biofilm formation and antibiotic biosynthesis (Suh et al., 1999; Liu et al., 2016; Qin et al., 2020). This present study showed that RpoS plays a pleiotropic role in the control of biocontrol-associated traits (Figure 8). RpoS is required for bacterial swimming and swarming motilities, and biofilm formation. This suggests that colonization ability of FD6 may be impaired and need further study. Our results also showed that RpoS is involved in stress resistance, suggesting decreased ability of *rpoS* mutant to survive in different environmental stresses. In addition, the RpoS mutation resulted in overproduction of antibiotics 2,4-DAPG and Plt, and improved antimicrobial activity of FD6.

RpoS has been well reported as a global regulator of general stress resistance in *E. coli*, *Pseudomonas* spp. and *Serratia plymuthica* (Heeb et al., 2005; Liu et al., 2016, 2018a). Similarly, our results demonstrated that a *rpoS* mutation led to higher sensitivity to ethanol, oxidative stress and heat shock, while osmotic stress was independent of RpoS in *P. protegens* FD6. Both *P. protegens* FD6 and spoilage bacterium *P. fluorescens* UK4 are similar in the resistance of RpoS to environmental stresses (Liu et al., 2018a). RpoS has been well characterized as a contributor to NaCl stress resistance in a number of pseudomonads (Suh et al., 1999; Wu et al., 2021); however, our data indicated that RpoS does not affect osmotic stress. Thus, the role of RpoS appears to vary in different *Pseudomonas* species.

Kidarsa et al. (2013) studied the transcriptome between *P. protegens* Pf-5 and its *rpoS* mutant on seeds and identified 476 genes under the control of RpoS. These genes are involved in iron homeostasis, secondary metabolism, cyclic diguanylate (c-di-GMP) signaling, biofilm formation, and antibiotic production. The RpoS mutation could alter the synthesis of the antifungal secondary metabolites pyrrolnitrin, pyoluteorin, phenazines and 2,4-DAPG (Sarniguet et al., 1995; Yan et al., 2009; He et al., 2019). The RpoS mutation in *P. protegens* FD6 also led to the increased yields of 2,4-DAPG and Plt. To understand whether yield changes in antibiotics correlate with related gene expression, the gene expression were compared in both the exponential and stationary phases of FD6 and the *rpoS* mutant. The qPCR data suggested that there was no significant difference both *phlA* and *pltA* expression in the ΔrpoS compared to that in the WT during the exponential growth phase. However, the expression of *phlA* and *pltA* was significantly changed (*p* < 0.001) during the stationary phase of ΔrpoS compared to that in FD6 (Supplementary Figure S3). Herein, we compared the transcript levels of the *phlHGFACBD* and *pltMRLABCDEFGZ* operons of FD6 and ΔrpoS during the stationary growth phase. The antibiotic 2,4-DAPG and Plt biosynthetic gene clusters were upregulated in the ΔrpoS, which was in accordance with the overproduction of 2,4-DAPG and Plt in the ΔrpoS mutant. In particular, the biosynthetic genes *pltMRLABCDEFGZ* for Plt were highly controlled by RpoS, with *pltA* showing the greatest increase (−5.6-fold) in the ΔrpoS mutant.

Based on our data, we hypothesize that RpoS serves as a repressor in antibiotic production. The role of RpoS as a negative regulator of antibiotic genes was also revealed in other *Pseudomonas* species (Yan et al., 2009; Kidarsa et al., 2013; He et al., 2019). However, the potential repressive mechanism of RpoS in antibiotic production remains unclear in the biological control agent *Pseudomonas* spp. To reveal the regulatory model by which RpoS functions in biocontrol agent FD6, the antibiotic 2,4-DAPG and Plt gene clusters were searched for the *Salmonella enterica* serovar Typhimurium RpoS binding motif (CTANNNT). We identified five putative RpoS binding motifs in the promoter regions of antibiotic-associated genes (*phlG*, *phlA*, *pltR*, *pltL* and *pltF*). PhlG hydrolase is associated with converting 2,4-DAPG to MAPG (Bottiglieri and Keel, 2006). However, RpoS does not appear to be the exclusive regulon of *phlG*. Another global transcriptional regulator, Vfr, positively regulates *phlG* expression *via* direct binding to an DNA sequence in the *phlG* promoter region (Zhang et al., 2021b). The TetR family regulator *phlH* also binds to the promoter region of *phlG* to repress its transcription (Yan et al., 2017b). Multiple transcriptional factors present in the genome of *P. protegens* FD6 may contribute to this strain to survive in diverse environments. pltR, a lysR-type transcriptional regulator, plays a transcriptional activator role in the Plt operon *pltLABCDEFG*. Plt has been reported as a signal to activate the expression of PltR (Li et al., 2012). Moreover, PG-Cl_2_ is necessary for the activation of *pltL* expression mediated by PltR (Yan et al., 2017a). Pyoluteorin biosynthesis begins with the activation of L-proline by the PltF and its parter PltL carrier protein (Thomas et al., 2002). Lévi-Meyrueis et al. (2015) suggested that *rpoS* mutants lacking promoter DNA binding lost their repressor activity based on global genome expression analysis, indicating that DNA binding plays a crucial role in the downregulation of gene expression by RpoS. Our data suggest that the DNA binding activity of RpoS is required for the downregulation of *phlA* and *pltL*. Further experiments will be performed to understand how the interactions among RpoS, PhlG, PhlA, PltR, PltL and PltF have influence on the synthesis of Plt and 2,4-DAPG. Here we reveal that RpoS binds directly to the promoter regions of *phlA*, *phlG*, *pltR*, *pltL* and *pltF*, thereby promoting RpoS-regulated repressive role in 2,4-DAPG and pyoluteorin biosynthesis.

This work focused on the functional analysis of the stationary-phase sigma factor RpoS in the biological agent *P. protegens* FD6. The results obtained from this study revealed that RpoS is involved in stress survival, biofilm formation and bacterial motility and mediates the biosynthesis of the antifungal metabolites 2,4-DAPG and Plt at both the transcriptional and translational levels. Moreover, transcription of the antibiotic biosynthetic genes *phlG*, *phlA*, *pltR*, *pltL* and *pltF* is under the control of RpoS, which directly binds to their promoter regions and inhibits the transcription initiation of these genes. This is the first report that DNA binding of RpoS is crucial for the suppression of 2,4-DAPG and Plt biosynthesis.

## Data availability statement

The datasets presented in this study can be found in online repositories. The names of the repository/repositories and accession number(s) can be found in the article/Supplementary material.

## Author contributions

QZ, JX, and QL designed the research. QZ wrote the manuscript article. ZX analyzed data. SL, YY, CX, and DW conducted the experiments. All authors contributed to the article and approved the submitted version.

## Funding

This work was supported by the National Natural Science Foundation of China (32072471, 31772210), Jiangsu Provincial Key Project for Science and Technology (2019338).

## Conflict of interest

The authors declare that the research was conducted in the absence of any commercial or financial relationships that could be construed as a potential conflict of interest.

## Publisher’s note

All claims expressed in this article are solely those of the authors and do not necessarily represent those of their affiliated organizations, or those of the publisher, the editors and the reviewers. Any product that may be evaluated in this article, or claim that may be made by its manufacturer, is not guaranteed or endorsed by the publisher.

## References

[ref1] Achkar J. Xian M. Zhao H. Frost J. W. (2005). Biosynthesis of phloroglucinol. J. Am. Chem. Soc. 127, 5332–5333. doi: 10.1021/ja042340g 15826166

[ref2] Bangera M. G. Thomashow L. S. (1999). Identification and characterization of a gene cluster for synthesis of the polyketide antibiotic 2,4-diacetylphloroglucinol from *Pseudomonas fluorescens* Q2-87. J. Bacteriol. 181, 3155–3163. doi: 10.1128/jb.181.10.31-55-3163.1999, PMID: 10322017 PMC93771

[ref3] Blumer C. Heeb S. Pessi G. Haas D. (1999). Global GacA-steered control of cyanide and exoprotease production in *Pseudomonas fluorescens* involves specific ribosome binding sites. Proc. Natl. Acad. Sci. U. S. A. 96, 14073–14078. doi: 10.1073/pnas.96.24.14073, PMID: 10570200 PMC24192

[ref4] Bottiglieri M. Keel C. (2006). Characterization of PhlG, a hydrolase that specifically degrades the antifungal compound 2,4-diacetylphloroglucinol in the biocontrol agent *Pseudomonas fluorescens* CHA0. Appl. Environ. Microbiol. 72, 418–427. doi: 10.1128/aem.72.1.418-427.2006, PMID: 16391073 PMC1352262

[ref5] Chang L. Li Q. Tong Y. H. Xu J. Y. Zhang Q. X. (2011). Identification of the biocontrol bacterial strain FD6 and antimicrobial study of this bacterium against tomato grey mould pathogen Botrytis cinerea. Acta. Phytophy. Sin. 38, 4874–4892. doi: 10.13802/j.cnki.Zwbhx-b.2011.06.002

[ref6] Dwivedi D. Johri B. N. (2003). Antifungals from fluorescent pseudomonads: biosynthesis and regulation. Curr. Sci. 85, 1693–1703. doi: 10.2307/24109974

[ref7] Girard G. van Rij E. T. Lugtenberg B. Bloemberg G. V. (2006). Regulatory roles of psrA and rpoS in phenazine-1-carboxamide synthesis by *Pseudomonas chlororaphis* PCL1391. Microbiology 152, 43–58. doi: 10.1099/mic.0.28284-0, PMID: 16385114

[ref8] Hall C. W. Hinz A. J. Gagnon L. B. Zhang L. Nadeau J. P. Copeland S.. (2018). *Pseudomonas aeruginosa* biofilm antibiotic resistance gene ndvB expression requires the RpoS stationary-phase sigma factor. Appl. Environ. Microbiol. 84, e02762–e02717. doi: 10.1128/aem.02762-17 29352081 PMC5861823

[ref9] Hanahan D. (1983). Studies on transformation of *Escherichia coli* with plasmids. J. Mol. Biol. 166, 557–580. doi: 10.1016/S0022-2836(83)80284-8 6345791

[ref10] He Q. Feng Z. Wang Y. Wang K. Zhang K. Kai L.. (2019). LasR might act as an intermediate in overproduction of phenazines in the absence of RpoS in *Pseudomonas aeruginosa*. J. Microbiol. Biotechnol. 29, 1299–1309. doi: 10.4014/jmb.1904.0402, PMID: 31387340

[ref11] Heeb S. Itoh Y. Nishijyo T. Schnider U. Keel C. Wade J.. (2000). Small, stable shuttle vectors based on the minimal pVS1 replicon for use in gram-negative, plant-associated bacteria. Mol. Plant. Microbe. Interact. 13, 232–237. doi: 10.1094/mpmi.2000.13.2.232, PMID: 10659714

[ref12] Heeb S. Valverde C. Gigot-Bonnefoy C. Haas D. (2005). Role of the stress sigma factor RpoS in GacA/RsmA-controlled secondary metabolism and resistance to oxidative stress in *Pseudomonas fluorescens* CHA0. FEMS Microbiol. Lett. 243, 251–258. doi: 10.1016/j.femsle.2004.12.008, PMID: 15668026

[ref13] Hengge-Aronis R. (2000). “The general stress response in *Escherichia coli*” in Bacterial stress responses. eds. Storz G. Hengge-Aronis R. (Washington, DC: ASM Press)

[ref14] Keswani C. Singh H. B. García-Estrada C. Caradus J. He Y. W. Mezaache-Aichour S.. (2020). Antimicrobial secondary metabolites from agriculturally important bacteria as next-generation pesticides. Appl. Microbiol. Biotechnol. 104, 1013–1034. doi: 10.1007/s00253-019-10300-8, PMID: 31858191

[ref15] Kidarsa T. A. Shaffer B. T. Goebel N. C. Roberts D. P. Buyer J. S. Johnson A.. (2013). Genes expressed by the biological control bacterium *Pseudomonas protegens* Pf-5 on seed surfaces under the control of the global regulators GacA and RpoS. Environ. Microbiol. 15, 716–735. doi: 10.1111/1462-2920.12066, PMID: 23297839

[ref16] Kill K. Binnewies T. T. Sicheritz-Pontén T. Willenbrock H. Hallin P. F. Wassenaar T. M.. (2005). Genome update: sigma factors in 240 bacterial genomes. Microbiology 151, 3147–3150. doi: 10.1099/mic.0.28339-0, PMID: 16207898

[ref17] Kovach M. E. Elzer P. H. Hill D. S. Robertson G. T. Farris M. A. Roop R. M.. (1995). Four new derivatives of the broad-host-range cloning vector pBBR1MCS, carrying different antibiotic-resistance cassettes. Gene 166, 175–176. doi: 10.1016/0378-1119(95)00584-1, PMID: 8529885

[ref18] Lévi-Meyrueis C. Monteil V. Sismeiro O. Dillies M. A. Kolb A. Monot M.. (2015). Repressor activity of the RpoS/σS-dependent RNA polymerase requires DNA binding. Nucleic Acids Res. 43, 1456–1468. doi: 10.1093/nar/gku1379, PMID: 25578965 PMC4330354

[ref19] Li S. Huang X. Wang G. Xu Y. (2012). Transcriptional activation of pyoluteorin operon mediated by the LysR-type regulator PltR bound at a 22 bp lys box in *Pseudomonas aeruginosa* M18. PLoS One 7:e39538. doi: 10.1371/journal.pone.0039538, PMID: 22761817 PMC3382589

[ref20] Ligon J. Hill D. Hammer P. Torkewitz N. Hofmann D. Kempf H.. (2000). Natural products with antifungal activity from pseudomonas biocontrol bacteria. Pest Manag. Sci. 56, 688–695. doi: 10.1533/9781845698416.4.179

[ref21] Liu X. Ji L. Wang X. Li J. Zhu J. Sun A. (2018a). Role of RpoS in stress resistance, quorum sensing and spoilage potential of *Pseudomonas fluorescens*. Int. J. Food Microbiol. 270, 31–38. doi: 10.1016/j.ijfoodmicro.2018.02.011, PMID: 29471265

[ref22] Liu Y. Shi H. Wang Z. Huang X. Zhang X. (2018b). Pleiotropic control of antibiotic biosynthesis, flagellar operon expression, biofilm formation, and carbon source utilization by RpoN in *Pseudomonas protegens* H78. Appl. Microbiol. Biotechnol. 102, 9719–9730. doi: 10.1007/s00253-018-9282-0, PMID: 30128583

[ref23] Liu X. Wu Y. Chen Y. Xu F. Halliday N. Gao K.. (2016). RpoS differentially affects the general stress response and biofilm formation in the endophytic *Serratia plymuthica* G3. Res. Microbiol. 167, 168–177. doi: 10.1016/j.resmic.2015.11.003, PMID: 26671319

[ref24] Liu X. Xu J. Zhu J. Du P. Sun A. (2019). Combined transcriptome and proteome analysis of RpoS regulon reveals its role in spoilage potential of *Pseudomonas fluorescens*. Front. Microbiol. 10:94. doi: 10.3389/fmicb.2019.00094, PMID: 30787912 PMC6372562

[ref25] Livak K. J. Schmittgen T. D. (2001). Analysis of relative gene expression data using real-time quantitative PCR and the 2^−ΔΔCT^ method. Methods 25, 402–408. doi: 10.1006/meth.2001.1262 11846609

[ref26] Miller JH (1972). Experiments in Molecular Genetics. Cold Spring Harbor Laboratory Press: Cold Spring Harbor, NY. pp, 352–355.

[ref27] Mishra J. Arora N. K. (2018). Secondary metabolites of fluorescent pseudomonads in biocontrol of phytopathogens for sustainable agriculture. Appl. Soil Ecol. 125, 35–45. doi: 10.10166/j.apsoil.2017.12.004

[ref28] Mulet M. Lalucat J. García-Valdés E. (2010). DNA sequence-based analysis of the *Pseudomonas* species. Environ. Microbiol. 12, 1513–1530. doi: 10.1111/j.1462-2920.2010.02181.x 20192968

[ref29] Nowak-Thompson B. Chaney N. Wing J. S. Gould S. J. Loper J. E. (1999). Characterization of the pyoluteorin biosynthetic gene cluster of *Pseudomonas fluorescens* Pf-5. J. Bacteriol. 181, 2166–2174. doi: 10.1128/jb.181.7.2166-2174.1999, PMID: 10094695 PMC93630

[ref30] O'Toole G. A. Pratt L. A. Watnick P. I. Newman D. K. Weaver V. B. Kolter R. (1999). Genetic approaches to study of biofilms. Method. Enzymol. 310, 91–109. doi: 10.1016/s0076-6879(99)10008-9 10547784

[ref31] Qin H. Liu Y. Cao X. Jiang J. Lian W. Qiao D.. (2020). RpoS is a pleiotropic regulator of motility, biofilm formation, exoenzymes, siderophore and prodigiosin production, and trade-off during prolonged stationary phase in *Serratia marcescens*. PLoS One 15:e0232549. doi: 10.1371/journal.pone.0232549, PMID: 32484808 PMC7266296

[ref32] Reimmann C. Beyeler M. Latifi A. Winteler H. Foglino M. Lazdunski A.. (1997). The global activator GacA of *Pseudomonas aeruginosa* PAO positively controls the production of the autoinducer N-butyryl-homoserine lactone and the formation of the virulence factors pyocyanin, cyanide, and lipase. Mol. Microbiol. 24, 309–319. doi: 10.1046/j.1365-2958.1997.3291701.x, PMID: 9159518

[ref33] Sal G. Manfioletti G. Schneider C. (1988). A one-tube plasmid DNA mini-preparation suitable for sequencing. Nucleic Acids Res. 16:9878. doi: 10.1093/nar/16.20.9878, PMID: 3186460 PMC338806

[ref34] Sambrook J. Russell D. W. (2001). Molecular Cloning: A Laboratory Manual, 3rd Edn, New York: Cold Spring Laboratory Press. pp, 55–60.

[ref35] Sarniguet A. Kraus J. Henkels M. D. Muehlchen A. M. Loper J. E. (1995). The sigma factor sigma s affects antibiotic production and biological control activity of *Pseudomonas fluorescens* Pf-5. Proc. Natl. Acad. Sci. U. S. A. 92, 12255–12259. doi: 10.1073/pnas.92.26.12255, PMID: 8618880 PMC40335

[ref36] Schnider-Keel U. Seematter A. Maurhofer M. Blumer C. Duffy B. Gigot-Bonnefoy C.. (2000). Autoinduction of 2,4-diacetylphloroglucinol biosynthesis in the biocontrol agent *Pseudomonas fluorescens* CHA0 and repression by the bacterial metabolites salicylate and pyoluteorin. J. Bacteriol. 182, 1215–1225. doi: 10.1128/jb.182.5.1215-1225.2000, PMID: 10671440 PMC94405

[ref37] Schuster M. Hawkins A. C. Harwood C. S. Greenberg E. P. (2004). The *Pseudomonas aeruginosa* RpoS regulon and its relationship to quorum sensing. Mol. Microbiol. 51, 973–985. doi: 10.1046/j.1365-2958.2003.03886.x, PMID: 14763974

[ref38] Simon R. Priefer U. Pühler A. (1983). A broad host range mobilization system for in vivo genetic engineering: transposon mutagenesis in gram negative bacteria. Nat. Biotechnol. 1, 784–791. doi: 10.1038/nbt1183-784

[ref39] Suh S. J. Silo-Suh L. Woods D. E. Hassett D. J. West S. E. Ohman D. E. (1999). Effect of rpoS mutation on the stress response and expression of virulence factors in *Pseudomonas aeruginosa*. J. Bacteriol. 181, 3890–3897. doi: 10.1128/jb.181.13.3890-3897.1999, PMID: 10383954 PMC93876

[ref40] Taguchi F. Ichinose Y. (2013). Virulence factor regulator (Vfr) controls virulence-associated phenotypes in *Pseudomonas syringae* pv. *tabaci* 6605 by a quorum sensing-independent mechanism. Mol. Plant Pathol. 14, 279–292. doi: 10.1111/mpp.12003, PMID: 23145783 PMC6638821

[ref41] Thomas M. G. Burkart M. D. Walsh C. T. (2002). Conversion of L-proline to pyrrolyl-2-carboxyl-S-PCP during undecylprodigiosin and pyoluteorin biosynthesis. Chem. Biol. 9, 171–184. doi: 10.1016/s1074-5521(02)00100-x, PMID: 11880032

[ref42] Wong G. T. Bonocora R. P. Schep A. N. Beeler S. M. Fong A. J. Shull L. M.. (2017). The genome-wide transcriptional response to varying RpoS levels in *Escherichia coli* K-12. J. Bacteriol. 199, e00755–e00716. doi: 10.1128/jb.00755-16 28115545 PMC5350281

[ref43] Wu P. Wang Z. Zhu Q. Xie Z. Mei Y. Liang Y.. (2021). Stress preadaptation and overexpression of rpoS and hfq genes increase stress resistance of *Pseudomonas fluorescens* ATCC13525. Microbiol. Res. 250:126804. doi: 10.1016/j.micres.2021.126804, PMID: 34144508

[ref44] Xu H. Chen H. Shen Y. Du L. Chou S. H. Liu H.. (2016). Direct regulation of extracellular chitinase production by the transcription factor LeClp in *Lysobacter enzymogenes* OH11. Phytopathology 106, 971–977. doi: 10.1094/phyto-01-16-0001-r, PMID: 27385597

[ref45] Yan Q. Philmus B. Chang J. H. Loper J. E. (2017a). Novel mechanism of metabolic coregulation coordinates the biosynthesis of secondary metabolites in *Pseudomonas protegens*. elife 6:e22835. doi: 10.7554/elife.22835, PMID: 28262092 PMC5395296

[ref46] Yan Q. Wu X. G. Wei H. L. Wang H. M. Zhang L. Q. (2009). Differential control of the PcoI/PcoR quorum-sensing system in *Pseudomonas fluorescens* 2P24 by sigma factor RpoS and the GacS/GacA two-component regulatory system. Microbiol. Res. 164, 18–26. doi: 10.1016/j.micres.2008.02.001, PMID: 18395434

[ref47] Yan X. Yang R. Zhao R. X. Han J. T. Jia W. J. Li D. Y.. (2017b). Transcriptional regulator PhlH modulates 2,4-diacetylphloroglucinol biosynthesis in response to the biosynthetic intermediate and end product. Appl. Environ. Microbiol. 83, e01419–e01417. doi: 10.1128/aem.01419-17 28821548 PMC5648904

[ref48] Zhang Q. Ji Y. Qi X. Chng S. Tong Y. Chen X.. (2016). Role of Vfr in the regulation of antifungal compound production by *Pseudomonas fluorescens* FD6. Microbiol. Res. 188-189, 106–112. doi: 10.1016/j.micres.2016.04.013, PMID: 27296968

[ref49] Zhang Q. X. Kong X. W. Li S. Y. Chen X. J. Chen X. J. (2020). Antibiotics of *Pseudomonas protegens* FD6 are essential for biocontrol activity. Australas. Plant. Path. 49, 307–317. doi: 10.1007/s13313-020-00696

[ref50] Zhang Q. Stummer B. E. Guo Q. Zhang W. Zhang X. Zhang L.. (2021a). Quantification of *Pseudomonas protegens* FD6 and *Bacillus subtilis* NCD-2 in soil and the wheat rhizosphere and suppression of root pathogenic *Rhizoctonia solani* AG-8. Biol. Control 154:104504. doi: 10.1016/j.biocontrol.2020.104504

[ref51] Zhang Q. Xiao Q. Xu J. Tong Y. Wen J. Chen X.. (2015). Effect of retS gene on antibiotics production in *Pseudomonas fluorescens* FD6. Microbiol. Res. 180, 23–29. doi: 10.1016/j.micres.2015.07.005, PMID: 26505308

[ref52] Zhang Q. Xing C. Kong X. Wang C. Chen X. (2021b). ChIP-seq analysis of the global regulator Vfr reveals novel insights into the biocontrol agent *Pseudomonas protegens* FD6. Front. Microbiol. 12:667637. doi: 10.3389/fmicb.2021.667637, PMID: 34054776 PMC8160232

