# Supplementary material for: Regulatory roles of RpoS in the biosynthesis of antibiotics 2,4-diacetyphloroglucinol and pyoluteorin of Pseudomonas protegens FD6
Source: Front Microbiol. 2022 Dec 8;13:993732. doi: 10.3389/fmicb.2022.993732 (PMC9793710; doi:10.3389/fmicb.2022.993732)
Supplement: Supplementary file 1 [file Data_Sheet_1.docx]

**Supplementary Material**

**Supplementary Tables**

**Table S1.** Bacterial strains and plasmids

| Strain or plasmid | Characteristics | Reference or source |
| --- | --- | --- |
| Strains |  |  |
| *E. coli* |  |  |
| DH5α | F^-^ *recA1 endA1 hsdR17 deoR thi-1 supE44 gyrA96*  *relA1*(*lacZYA-argF*)*U169ë-*(*Ö80dlacz △M15*) | Hanahan et al. 1983 |
| S17-1 | *recA pro hsdR- M^+^RP4 2-Tc::Mu-Km::Tn7 Sm^r^ /Tp^r^* | Simon et al. 1983 |
| BL21 | *F^-^ ompT, hsdSB (rB^-^ mB^-^), gal, dcm met* (DE3) | Novagen |
| XL1-Blue MRF’ Kan | *E. coli* cloning strain; Km^r^ | Guo et al. 2009 |
| pTvfr-pBvfr | Bacterial one-hybrid positive control | Zhang et al. 2021b |
| *P. protegens* FD6 | Wild type; 2,4-DAPG^+^, Plt^+^, Ap^r^ | Chang et al. 2011 |
| ΔrpoS | *rpoS* in-frame deletion in strain FD6; Ap^r^ | This study |
| ΔrpoSC | ΔrpoS containing intact *rpoS* ORF; Km^r^ | This study |
| ΔrpoS_M_ | ΔrpoS containing *rpoS*_R147S_; Km^r^ | This study |
| *Botrytis cinerea* | The fungal pathogen causing tomato gray mold | Lab collection |
| *Monilinia fructicola* | The fungal pathogen causing peach brown rot | Lab collection |
| *Phytophthora capsici* | The fungal pathogen causing pepper blight | Lab collection |
| *Ralstonia solanacearum* | The fungal pathogen causing tomato bacterial wilt | Lab collection |
| Plasmids |  |  |
| p2P24 | Suicide plasmid for generation of gene in-frame deletions; *SacB*; Km^r^ | Yan et al. 2017b |
| pBBR1MCS-2 | Broad-host-range cloning vector; Km^r^ | Kovach et al. 1995 |
| pME6522 | pVS1-p15A shuttle vector for transcriptional *lacZ* fusions; Tet^r^ | Blumer et al. 1999 |
| pME6013 | pVS1-p15A shuttle vector for translational *lacZ* fusions; Tet^r^ | Heeb et al. 2000 |
| pET22b（+） | f1 origin, expression vector; Ap^r^ | Novagen |
| pTRG | The plasmid used for protein expression in bacterial one-hybridization assay; Tet^r^ | Stratagene |
| pBXcmT | Plasmid used for DNA cloning in the bacterial one-hybrid assay, Cm^r^ | Guo et al. 2009 |
| pBBR-rpoS | pBBR1MCS-2 carrying intact *rpoS* gene; Km^r^ | This study |
| p2P24-ΔrpoS | p2P24 derivative for generation of *rpoS* deletion, *SacB*; Km^r^ | This study |
| pTRG-rpoS | pTRG with the coding region of *rpoS*; Tet^r^ | This study |
| pBXcmT-pltR | pBXcmT with putative RpoS binding sites of *pltR*; Cm^r^ | This study |
| pBXcmT-pltF | pBXcmT with putative RpoS binding sites of *pltF*; Cm^r^ | This study |
| pBXcmT-pltL | pBXcmT with putative RpoS binding sites of *pltL*; Cm^r^ | This study |
| pBXcmT-phlA | pBXcmT with putative RpoS binding sites of *phlA*; Cm^r^ | This study |
| pBXcmT-phlG | pBXcmT with putative RpoS binding sites of *phlG*; Cm^r^ | This study |
| 6522-phlA | pME6522 containing 468 bp fragment of the promoter region of *phlA* gene; Tet^r^ | This study |
| 6522-pltL | pME6522 containing 560 bp fragment of the promoter region of *pltL* gene; Tet^r^ | This study |
| 6013-phlA | pME6013 containing 468 bp fragment of the promoter region of *phlA* gene; Tet^r^ | This study |
| 6013-pltL | pME6013 containing 560 bp fragment of the promoter region of *pltL* gene; Tet^r^ | This study |
| pET22b-rpoS | pET22b with 1005 bp fragment including *rpoS*; Ap^r^ | This study |
| pMD19-T | Clone vector; Ap^r^ | TaKaRa |
| pMD-rpoS | pMD19-T containing 1008 bp *rpoS*; Ap^r^ | This study |
| pMD-rpoS_R147S_ | pMD-rpoS derivative for generation of *rpoS* point mutation; Ap^r^ | This study |
| pBBR-rpoS_R147S_ | pBBR derivative for generation of *rpoS* point mutation; Km^r^ | This study |

Ap^r^, ampicillin resistant; Km^r^, kanamycin resistant; Gm^r^, gentamycin resistant; Tet^r^, tetracycline resistant.

| **Table S2.** Primer and oligonucleotide sequences | | |  | | |
| --- | --- | --- | --- | --- | --- |
| Primer name | | Sequence (5’ →3’)^a^ | | Amplicon (bp) | |
| **Primers used for construction of vector** | | | |  | |
| **RpoS deletion** | |  | |  | |
| ropS422-F1 | | CAT**GAGCTC**AATGAGTGGGACTGCGATC | | 1273 | |
| ropS1695-R1 | | CAAGGATCTCTCGCAGGCGTTTGAGGAG | |  |  |
|  | | GGCACGCGTGTAATCAATGTAC | |  | |
| ropS2482-F2 | | GTACATTGATTACACGCGTGCCCTCCTC | | 656 | |
|  | | AAACGCCTGCGAGAGATCCTTG | |  |  |
| ropS3138-R2 | | ACG**TCTAGA**GATCTGGCCGAATATCAC | |  |  |
| **RpoS complementation** | |  | |  | |
| ropS1239-F | | CGG**GGTACC**GGCGAATTAGTCATCATC | | 1429 | |
| ropS2650-R | | ACG**GAGCTC**ATCCCAAAGCTCAGAATC | |  |  |
| **Transcriptional and translational fusions** | | | |  | |
| phlA6013-F-440 | | TA**GAATTC**CAAACGCAACGCAGGGTATAGG | | 468 | |
| phlA6013-R27 | | TC**GGATCC**CCATAGCTGACAATGCCTACT | |  |  |
| pltL6013-57F | | AG**GAATTC**GTTTGTCAAGCAGTGCTGG | | 560 | |
| pltL6013-616R | | AC**GGATCC**CCACCGATAATCACTAC | |  |  |
| **Prokaryotic expression** | |  | |  | |
| RpoS-NdeI-F | | CGC**CATATG**GCTCTCAGTAAAGAAGT | | 1008 | |
| RpoS-XhoI-R | | CCG**CTCGAG**CTGAAATAGAGACTCACTGG | |  |  |
| **Site mutation** | |  | |  | |
| RpoS-mut-pBBR-F | | **GTCGAC**ATGGCTCTCAGTAAAGAAGT | | 1008 | |
| RpoS-mut-pBBR-R | | **TCTAGA**CTACTGAAATAGAGACTCATCGG | |  |  |
| RpoS-mut-F | | CCGGAACGCGGCTTCAGCTTTTCGA | | 4008 | |
| RpoS-mut-R | | TGAAGCCGCGTTCCGGGTCGAACTT | |  |  |
| RpoS M-F | | CTACTGAAATAGAGACTCACTG | | 1008 | |
| RpoS M-R | | CGGAATTCAGGCCTGTATGACGATCTTC | |  |  |
| **Primers used for bacterial one-hybrid** | | | |  | |
| phlGp-F | | CG**GAATTC**ATCTGCTTGCTGGTGGTGTT | | 395 | |
| phlGp-R | | GC**TCTAGA**GAGCGGAAATACTTGGCG | |  |  |
| phlAp-F | | CG**GAATTC**CTCCGAATCGAAATGAAA | | 566 | |
| phlAp-R | | GC**TCTAGA**TTCATCGGGCTGCAGCAC | |  |  |
| pltRp-F | | CG**GAATTC**AGGCCTGTATGACGATCTTC | | 576 | |
| pltRp-R | | GC**TCTAGA**ACTTCTGATCTAGCATGCC | |  |  |
| pltLp-F | | CG**GAATTC**TAGTTCTAGTGGCCAATTGC | | 345 | |
| pltLp-R | | GC**TCTAGA**GGAGTCTGGTCATCAAGCTC | |  |  |
| pltFp-F | | CG**GAATTC**CAGCTCAACATTGCCCTGTC | | 334 | |
| pltFp-R | | GC**TCTAGA**CAGAGCATTGCCACGAATTC | |  |  |
| pTRG-RpoS-F | | CG**GAATTC**ATGGCTCTCAGTAAAGAAGT | | 1008 | |
| pTRG-RpoS-R | | CC**CTCGAG**CTACTGAAATAGAGACTCACTG | |  |  |
| ^a^ Boldfaced letters are restriction sites. | | | |  | |
| **Primers used for EMSA** | |  | |  | |
| phlG E-F | | CGCTCTCACTAAGTTAAATTCAAAT | | 25 | |
| phlG E-R | | TAAACTTAAATTGAATCACTCTCGC | |  |  |
| phlA E-F | | TTGCACCGATCTATCTAGTTTTTAA | | 25 | |
| phlA E-R | | AATTTTTGATCTATCTAGCCACGTT | |  |  |
| pltR E-F | | CCACTAGAACTATTTTTGTATCCAG | | 25 | |
| pltR E-R | | GACCTATGTTTTTATCAAGATCACC | |  |  |
| pltL E-F | | AACTATGCTTTGGCGCTCGAAAATG | | 25 | |
| pltL E-R | | GTAAAAGCTCGCGGTTTCGTATCAA | |  |  |
| pltF E-F | | CGCGATTCCATCGGCACTACGATCT | | 25 | |
| pltF E-R | | TCTAGCATCACGGCTACCTTAGCGC | |  |  |
| **Primer sequences for qPCR** | |  | |  | |
| rrsB-qpcr-F | | GGAAGGAACACCAGTGGCGA |  | | |
| rrsB-qpcr-R | | TCAAGGCTCCCAACGGCT |  | | |
| phlH-qpcr-F | | GCTGCGGGCGATCATCACTG |  | | |
| phlH-qpcr-R | | GCACCGACGAAGGCGACAG |  | | |
| phlG-qpcr-F | | GCATGTACCTGACCAGCCGTTAC |  | | |
| phlG-qpcr-R | | TCGCCAAAGCCACTTTCCTTGAG |  | | |
| phlF-qpcr-F | | CAACCGTGAATTGCTGCTGGATATG |  | | |
| phlF-qpcr-R | | AATGTCCTGTTCCGCCGTCAAC |  | | |
| phlA-qpcr-F | | GCCATCGAGCAGTACCAGAAGC |  | | |
| phlA-qpcr-R | | GGTAGGCGGTCAGGGCGTAG |  | | |
| phlC-qpcr-F | | CAGCAGTTCGGTGTCGTTCCAG |  | | |
| phlC-qpcr-R | | CTCGGCGTAGTTGAAGTGATCGG |  | | |
| phlB-qpcr-F | | CGAGCATGGCGGCAAGTACC |  | | |
| phlB-qpcr-R | | AAGGCTTCACGCTCAGGGAATTG |  | | |
| phlD-qpcr-F | | AACCTCCACGCCGACCATCC |  | | |
| phlD-qpcr-R | | GGCTTCACGCTCATAGACGATGC |  | | |
| phlE-qpcr-F | | GCCTCGATTGCCGTGCTGTC |  | | |
| phlE-qpcr-R | | CCTTCGCAGATGCCCATCAACC |  | | |
| pltM-qpcr-F | | TCCAGTTCAACAACGCCAAGTACC | | |  |
| pltM-qpcr-R | | GCAGCAGGCAGAAGCGATCC | | |  |
| pltR-qpcr-F | | GTACCAACATGCCTGCCTGATCC | | |  |
| pltR-qpcr-R | | TGCGTGGATGATTCCTATGCCTTG | | |  |
| pltL-qpcr-F | | GCACTCGGCCTTTAGTTGCT | | |  |
| pltL-qpcr-R | | CCAAGTACCCATATCACCGGC | | |  |
| pltA-qpcr-F | | GCCATCGAGCAGTACCAGAAGC | | |  |
| pltA-qpcr-R | | GGTAGGCGGTCAGGGCGTAG | | |  |
| pltB-qpcr-F | | GTGGATGCTCAGGTGCGAAGTG | | |  |
| pltB-qpcr-R | | CTGTTGCTGGATGCCGTGCTC | | |  |
| pltC-qpcr-F | | GGCTGGACCTGGACTTCTTTGC | | |  |
| pltC-qpcr-R | | AACTGATTGGCGGCTGCGTAG | | |  |
| pltD-qpcr-F | | GGTGGTGCCGTTCGATGAGTG | | |  |
| pltD-qpcr-R | | TGCGAGTGCTGGTGGTTGTTG | | |  |
| pltE-qpcr-F | | TTGACGATTGTGTGGTTCCTGAGG | | |  |
| pltE-qpcr-R | | CCAGTTGCCGCTCCATCATCC | | |  |
| pltF-qpcr-F | | CGCAGTGGGTGAAGAAGGTGAG | | |  |
| pltF-qpcr-R | | CCAGGTATTCGTAGTTGCCGTCAG | | |  |
| pltG-qpcr-F | | CCTCGGTCTTTCATCGCTGGTTC | | |  |
| pltG-qpcr-R | | CGCCTGCATGTCGCTGTAGC | | |  |
| pltZ-qpcr-F | | TCACCAGCCTTGTGCAAGAAGAAG | | |  |
| pltZ-qpcr-R | | CGATCTATCACCGCCAGCATCAG | | |  |
| pltI-qpcr-F | | AAGATCGGCCCACGCAATGAAG | | |  |
| pltI-qpcr-R | | GTAATGTTCCAGCAGGTCCAGGTC | | |  |
|  |  |  |  |  |  |
|  |  |  |  |  |  |
|  |  |  |  |  |  |
|  |  |  |  |  |  |
|  |  |  |  |  |  |
|  |  |  |  |  |  |
|  |  |  |  |  |  |
|  |  |  |  |  |  |
|  |  |  |  |  |  |
|  |  |  |  |  |  |
