# Supplementary material for: Regulatory roles of RpoS in the biosynthesis of antibiotics 2,4-diacetyphloroglucinol and pyoluteorin of Pseudomonas protegens FD6
Source: Front Microbiol. 2022 Dec 8;13:993732. doi: 10.3389/fmicb.2022.993732 (PMC9793710; doi:10.3389/fmicb.2022.993732)
Supplement: Supplementary file 2 [file Image_1.PDF]

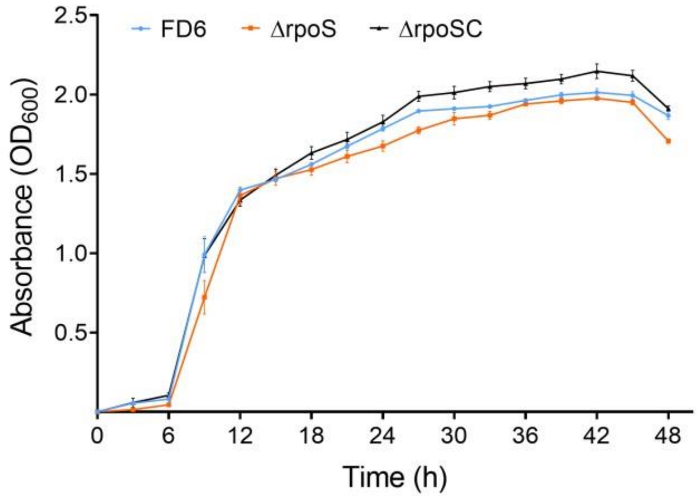

Figure S1. Growth curves of *P. protegens* FD6 and its derivatives in LB medium. All overnight cultures were adjusted to an OD_{600} of 0.5, diluted 1:100 into 50 mL LB broth, and grown at 28 °C with constant shaking. The absorbance was measured at 3-h intervals at 600 nm for 48 h. The data are presented as the mean±SD.
