# Supplementary material for: Regulatory roles of RpoS in the biosynthesis of antibiotics 2,4-diacetyphloroglucinol and pyoluteorin of Pseudomonas protegens FD6
Source: Front Microbiol. 2022 Dec 8;13:993732. doi: 10.3389/fmicb.2022.993732 (PMC9793710; doi:10.3389/fmicb.2022.993732)
Supplement: Supplementary file 3 [file Image_2.PDF]

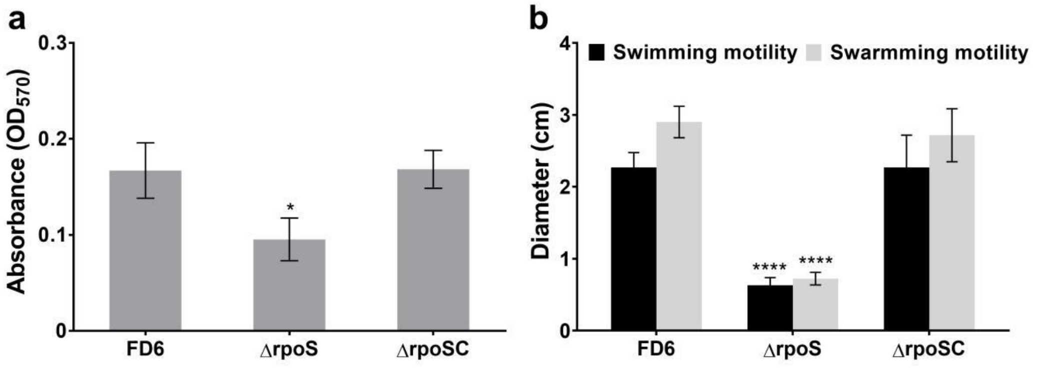

Figure S2. Phenotypic characterization of *P. protegens* FD6 strains. a: Quantification of biofilm formation by *P. protegens* strains. Overnight cultures were diluted 1:1000 in LB, transferred 500 μL into Eppendorf tubes, and incubated statically for 24 h. The adherent cells were stained with 0.1% crystal violet for 20 min and quantified at 570 nm. b: Impact of *rpoS* deletion on motility. Overnight cultures were inoculated on SWM swarm or MMMF swim plates and incubated for 48 h at 28 °C. Error bars indicate standard deviation (n=3). *P<0.05, ****P<0.0001.
