# Supplementary material for: Regulatory roles of RpoS in the biosynthesis of antibiotics 2,4-diacetyphloroglucinol and pyoluteorin of Pseudomonas protegens FD6
Source: Front Microbiol. 2022 Dec 8;13:993732. doi: 10.3389/fmicb.2022.993732 (PMC9793710; doi:10.3389/fmicb.2022.993732)
Supplement: Supplementary file 4 [file Image_3.pdf]

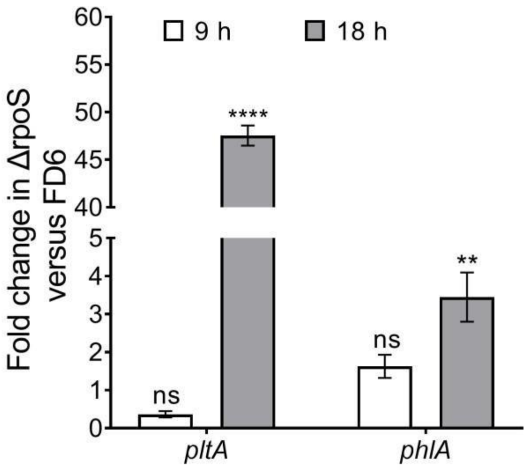

Figure S3. Regulation of *phlA* and *pltA* transcription by RpoS in different growth phases.Gene expression was determined by qRT–PCR. The graph shows fold changes in gene transcription of phlA and pltA in the RpoS mutant versus FD6. ****P < 0.0001.
